# Congenital myelomeningocele - do we have to change our management?

**DOI:** 10.1186/1743-8454-7-17

**Published:** 2010-10-14

**Authors:** Steffi Mayer, Margit Weisser, Holger Till, Gerd Gräfe, Christian Geyer

**Affiliations:** 1 Department of Pediatric Surgery, University Hospital Leipzig, Liebigstrasse 20a, 04103 Leipzig, Germany

## Abstract

**Background:**

Eagerly awaiting the results of the Management of Myelomeningocele Study (MOMS) and with an increasing interest in setting up intrauterine myelomeningocele repair (IUMR), the optimal management of patients suffering from congenital myelomeningocele (MMC) has become a matter of debate again. We performed a cross-sectional study at our referral-center for MMC to determine the outcome for our expectantly managed patients.

**Materials and methods:**

A computed chart review at our institution revealed 70 patients suffering from MMC. Forty-three patients were eligible for the study and analyzed further. A retrospective analysis was performed only in patients that underwent MMC repair within the first two days of life and were seen at our outpatient clinic between 2008 and 2009 for a regular multidisciplinary follow-up. Data were collected on: gestational age (GA) and weight at birth, age at shunt placement and shunt status after the first year of life, radiological evidence for Arnold-Chiari malformation (ACM) and tethered cord (TC), need for surgery for TC, bladder function, lower leg function and educational level. Data were compared to published results for IUMR and to studies of historical controls.

**Results:**

Patients were born with MMC between 1979 and 2009 and are now 13.3 ± 8.9 (mean ± SD) years of age. At birth, mean GA was 37.8 ± 2.3 weeks and mean weight was 2921.3 ± 760.3 g, both significantly higher than in IUMR patients. Shunt placement in our cohort was required in 69.8% at a mean age of 16.0 ± 10.7 days, which was less frequent than for historical controls. Amongst our cohort, radiological observations showed 57.1% had ACM II and 41.9% had TC. Only two of our patients underwent a surgical correction for TC. Clean intermittent catheterization was performed in 69.7% of our patients, 56.4% were (assisted) walkers and 64.1% attended regular classes, both comparable to historical controls.

**Conclusions:**

With a close and interdisciplinary management by pediatric surgeons, neurologists and urologists, the long-term outcome of patients suffering from MMC can currently be considered satisfactory. With respect to the known drawbacks of fetal interventions for mother and child, especially preterm delivery, the results of the MOMS trial should be awaited with caution before proceeding with a complex intervention like IUMR.

## Background

Myelomeningocele (MMC) is a mostly isolated congenital disorder of the central nervous system that has a multifactorial etiology. Based on a prevalence of 10-15 per 10,000, more than 4,500 pregnancies are affected in the European Union each year [1]. Its prevalence can be reduced by 50-70% with maternal 400 μg folic acid supplementation before conception and during the first trimester [2,3]. Myelomeningocele is characterized by a protrusion of the meninges and spinal cord through open vertebral arches which results in varying degrees of paralysis, mental retardation, bowel and bladder dysfunction as well as orthopedic disabilities [4]. After surgical closure of the defect, many patients present with a hydrocephalus that requires the placement of a ventricular shunt to prevent additional cerebral damage, which again is associated with a high rate of complications like dysfunction and infection [5]. Most patients are further affected by an Arnold-Chiari malformation (ACM) due to a downward movement of the hindbrain and obstruction of the normal egress of cerebrospinal fluid (CSF) from the fourth ventricle that increases the 5-year-mortality from 7.9% to 35% [6-8]. A high number of patients also suffer from spinal cord tethering (TC), which progressively worsens neurological function and frequently requires surgical correction [9]. In 1990, the two-hit-hypothesis for the pathogenesis of MMC was postulated by Heffez *et al*: A defective spinal development is followed by an intrauterine injury of the spinal cord due to the exposure to amniotic fluid, meconium and urine, as well as direct trauma and hydrodynamic pressure, thus causing loss of neural tissue due to a progressive cell toxicity over gestation [10-13].

Spina bifida is nowadays diagnosed prenatally in 70-90% of cases mostly before 20 weeks of gestational age (GA) by routine ultrasound scan [14,15]. If the ultrasound scan is positive, amniocentesis is performed to rule out genetic syndromes and to measure alpha-fetoprotein (AFP) levels. Mostly, parents are counseled to opt for expectant management or termination of pregnancy. In the United States, an intrauterine myelomeningocele repair (IUMR) can also be offered. The concept of IUMR is based on the hypothesis that an intrauterine protection of the exposed spinal cord as well as the reduction of continuous intramniotic leakage of CSF might prevent some of its secondary damage [16,17]. Since the first intrauterine endoscopic repair in 1994, which was replaced by open surgical repair in 1997, about 400 open fetal interventions for MMC have now been performed worldwide [4]. Preliminary results suggest a reversal of hindbrain herniation, a decrease in shunt-dependent hydrocephalus, an improvement in leg function and an unaltered bladder function after IUMR [4,18,19]. However, the technique remains of unproven benefit and the reported findings might be explained by selection bias and changing management indications [20]. Therefore, the Management of Myelomeningocele Study (MOMS), a multicenter, prospective, randomized controlled trial of 200 patients (100 fetal repair, 100 postnatal repair), was set up in 2003 in the US (San Francisco, Nashville and Philadelphia) to primarily investigate death or the need for shunting by the age of one year with and without fetal intervention in MMC http://www.spinabifidamoms.com.

The objective of this study was to decide, whether it is time to offer IUMR at our institution to patients diagnosed prenatally with MMC. Therefore, we performed a retrospective data analysis on expectantly managed patients with MMC that attended our hospital and compared them to results obtained after IUMR at other institutions and to historical controls as collected from the literature.

## Methods

### Patient recruitment

A computed chart review was performed in February 2010 to conduct a descriptive and retrospective study on information obtained from medical reports. Patients were recruited to the study if they suffered from an open spina bifida that was diagnosed pre- or postnatally and underwent surgical repair within the first two days of life to prevent further damage, e.g. by CSF leakage, local infection or scarred shrinking, as generally accepted.

### Surgical repair of open spina bifida

After an ellipsoid incision of the *zona cutanea*, the *zona epithelioserosa *was dissected. The dura was mobilized completely and isolated from the *fascia thoracolumbalis*. The *zona epithelioserosa *was excised and the neural tube was reconstructed if possible using 10-0 absorbable single sutures. The dural layer was closed in craniocaudal direction using a 6-0 or 7-0 absorbable running suture, exceptionally inserting a dural patch. After the water-tight closure of the dura, the defect was covered by a wing-flap plasty. The *fascia thoracolumbalis *was dissected and closed before the subcutaneous tissue and skin layer were sutured. A primary stainless skin closure is essential in cases with extremely large defects, for which we preferred longitudinal incisions with mobilization of the skin and temporary skin substitution if necessary. Postoperative monitoring consisted of frequent clinical investigation, measurement of head circumference and cranial ultrasound examination.

### Shunt placement

A ventricular-peritoneal shunt was placed only in the case of symptomatic hydrocephalus, according to similar criteria to that listed in Table 1.

**Table 1:** Criteria for shunt placement

Criteria for Shunt placement (MOMS trial)	
*At least two of the following:*	*Or:*

An increase in the greatest occipitofrontal circumference adjusted for gestational age and defined as crossing percentiles	Head circumference > 95^th ^percentile for gestational age

A bulging fontanelle*, split sutures or sunsetting sign	Presence of marked syringomyelia (syrinx and expansion of spinal cord) and ventriculomegaly (undefined)

Increasing hydrocephalus on two consecutive imaging studies determined by an increase in ratio of biventricular diameter to biparietal diameter^§^	Ventriculomegaly (undefined) and symptoms of Chiari malformation (stridor, swallowing difficulties, apnea, bradycardia)

	Persistent cerebrospinal fluid leakage from the myelomeningocele wound or bulging at the repair site

Criteria for shunt placement as defined for the MOMS trial. *Bulging fontanelle: above the bone as assessed when the baby is in an upright position and not crying. ^§^Defined by O'Hayon *et al*, 1998 [34]. Table adapted from Tulipan *et al*, 2004 [35].

### Patient follow-up

All included patients were regularly seen at our outpatient clinic for a multidisciplinary follow-up, including consultation with a pediatric neurologist, pediatric urologist, pediatric orthopedists and pediatric surgeon. The final follow-up was between 2008 and 2009 in order to assess the patients' current health status.

### Data collection

Data were collected on: GA and weight at birth, neonatal death (defined as death within the first 28 days of life), shunt status at the first year of life and age at shunt placement, radiological presence of ACM and TC as assessed postnatally by ultrasound and/or magnetic resonance imaging, the need for surgery for TC, as well as bladder function, lower leg function and educational level. Satisfactory bladder function was defined as use of clean intermittent catheterization (CIC) and satisfactory educational level as regular attendance at kindergarten, school or job.

### Comparison with published data

We performed computerized bibliographic searches using Pubmed http://www.pubmed.gov and Embase http://www.embase.com databases to identify studies that report on outcome measurements after IUMR ("IUMR group") and for postnatally-managed patients with spina bifida ("historical controls"). If applicable, studies on which the MOMS trial had been based were preferentially included as historical controls. After identification of matching studies, data extraction was performed for outcomes as listed above. If the same outcome was published several times, e.g. mean gestational age at birth, the cohort with the largest number of patients was used for comparison.

### Study groups

The study consisted of three groups: (1) retrospectively assessed data on patients with open spina bifida that underwent MMC closure and follow-up at our institution ("Leipzig group"), (2) published data on patients that underwent IUMR elsewhere ("IUMR group") and (3) published data on patients that were operated postnatally and managed elsewhere, preferentially providing the basis for the MOMS trial ("historical controls") (Table 2).

**Table 2:** Characteristics of patients studied

Study Group	Reference	Publication Year	Study Site	Study Period	N patients	Level of lesion (%)			
						
						thoracic	lumbar	sacral	other
**(1)** **Leipzig group**			Leipzig, Germany	1979-2009	43	11.6	79.1	9.3	-

**(2)** **IUMR group**	Bruner [22]	1999	Vanderbilt, USA	1990-1997	29	median L4 (S-T12)			
	
	Tulipan [21]	2003	CHOP, Vanderbilt, USA	1983-2000	177	4.8	80.8	14.4	-
	
	Johnson [24]	2003	CHOP, USA	1998-2002	48	5	84	11	-
	
	Bruner [25]	2004	Vanderbilt, USA	1997-2003	116	4.3	81.9	13.8	-
	
	Brunner [23]	2005	Vanderbilt, USA	1997-2003	116	4.3	81.9	13.8	-
	
	Koh [27]	2006	Boston, USA	1979-2003	5	lumbosacral			
	
	Danzer [26]	2008	CHOP, USA	1998-2003	54	7.4	85.2	7.4	-
	
	Danzer [18]	2009	CHOP, USA	1998-2003	54	7.4	81.5	11.1	
	
	Danzer [29]	2010	CHOP, USA	1998-2003	30	median L4 (T12-S1)			

**(3) Historical controls**	Bruner [22]	1999	Vanderbilt, USA	1990-1997	23	median L4 (S-T12)			
	
	Bowman [5]	2001	Chicago, USA	1975-1979	71	35.2	42.2	21.2	1.4
	
	Tulipan [21]	2003	CHOP, Vanderbilt, USA	1983-2000	189	18.5	60.3	21.2	-
	
	Koh [27]	2006	Boston, USA	1979-2003	88	-			
	
	Aguilera [15]	2009	Bristol, UK	1999-2007	74	10.8	41.9	44.6	2.7

Summarized characteristics of included studies sorted by study group.

### Statistical analysis

Data collected from our cohort study ("Leipzig group") were analyzed assessing mean and standard deviation (SD) for continuous data (GA and weight at birth, age at shunt placement) and percentages for dichotomous data (neonatal death, birth before 30 weeks GA, shunt status at the first year of life, radiological presence of ACM II and TC, use of CIC, lower leg function and educational level). The collected results were compared to data published for IUMR and historical controls, respectively. For continuous data two-sided t-tests were applied to compare published means against sample means ("Leipzig group"). In none of the published articles were standard deviations presented. Therefore, similar standard deviations to our studies were used, assuming comparable distributions in similar populations. For nominal data contingency tables were generated from the total numbers of all study groups and tested applying two-sided Fisher's exact test. Regression analysis was further performed to test the correlation between birth year and prenatal diagnosis of our population. All statistical analyses were performed using JMP 7 software (SAS Institute, Cary, NC, USA). Data are given in raw numbers, percentage or mean ± SD if not indicated differently. Results were considered statistically significant at *p *< 0.05.

## Results

### Study population

The chart review revealed 70 patients suffering from spina bifida. Of those, two underwent IUMR elsewhere, five underwent MMC closure beyond the second day of life, 10 had not undergone surgery for MMC at our hospital or the operation date could not be identified and 10 patients were lost from follow-up. Therefore, data from 43 MMC patients that underwent surgical correction for MMC within the first two days of life and had been seen at our outpatient clinic between 06/2008 and 12/2009 were analyzed. All results are summarized in Table 3. The 43 patients were born between 1979 and 2009 and were on average 13.3 ± 8.9 y of age at the time of data analysis. 11.6% had thoracic, 79.1% lumbar and 9.3% sacral lesions (Table 2).

**Table 3:** Summarized results and statistical comparisons

	(1) Leipzig group	(2) IUMR group	(3) Historical controls	Statistical comparison (p-value)		
				
				(2) vs (3)	(1) vs (2)	(1) vs (3)
Neonatal death (%)	0	4.5 [21]	NA	-	< 0.05	-

GA at birth (wk)	37.8 ± 2.3	34.6 [21]	37.0 [22]	-	< 0.0001	ns
	
		33.2 [22]	37.0 [22]	< 0.001 [22]	-	-

GA < 30th wk (%)	0	11.8 [23]	NA	-	< 0.05	-

Birth weight (g)	2921.3 ± 760.3	2512 [24]	3075 [22]	-	< 0.01	ns
	
		2171 [22]	3075 [22]	< 0.001 [22]	-	-

Shunt placement ≤ 1 y (%)	69.8	54.3 [25]	85.7 [21]	< 0.0001	ns	< 0.05

Age at shunt placement (d)	16.0 ± 10.7	21.2 [24]	-	-	< 0.05	-
	
	12.5^$^	85.5 [22]^$^	5 [28]^$^	< 0.01 [22]	-	-

Incidence ACM II (%)	57.1	100^§^	75.7 [15]	-	-	ns

Surgery TC (%)	11.1	29.6 [26]	32.4 [5]	ns	< 0.01	< 0.001

(Assisted) Walkers (%)	56.4^£^	92.6 [18]^#^	59.2 [5]^¶^	< 0.0001	< 0.0001	ns

Wheelchair users (%)	41.0^£^	7.4 [18]^#^	40.8 [5]^¶^	< 0.0001	< 0.0001	ns

Regular education (%)	64.1	NA	63.4 [5]	-	-	ns

CIC (%)	69.7	NA	84.5 [5]	-	-	ns

Summarized results of our data collection compared to published results for historical controls and IUMR applying the two-sided t-test or Fisher's exact test at *p *< 0.05. Data are presented in mean (± SD) or median and percentages. GA: gestational age, ACM: Arnold-Chiari malformation, TC: tethered cord, CIC: clean intermittent catheterization. NA: not applicable, ns: not significant. ^$^median; ^§^ACM is one of the inclusion criteria for IUMR; ^£^mean age 13.3 ± 8.9 years, ^#^mean age 67.0 ± 18.2 months, ^¶^mean age 21.7 years.

### Prenatal diagnosis and birth

Only 37.2% of our patients had been diagnosed prenatally with MMC. However, the number of prenatal diagnoses significantly increased over time with a significant correlation between prenatal diagnosis and birth year (*p *< 0.0001). There were no neonatal deaths in our study population compared to 4.5% after IUMR (*p *< 0.05) [21]. Mean GA at birth in our population (37.8 ± 2.3 weeks) and of historical controls (37.0 weeks) was significantly higher than after IUMR (34.6 weeks; *p *< 0.001) without a significant difference between our population and historical controls [21,22]. None of our patients but 11.8% of the children after IUMR were born before 30 weeks of gestation (*p *< 0.05) [23]. Mean weight at birth in our group was 2921.3 ± 760.3 g, which was significantly higher than after IUMR (2512 g; *p *< 0.01) and not significantly different from historical controls (3075 g) [22,24].

### Shunt status

Among historical controls, median 85.7% of the patients required shunt placement within the first year of life, which was significantly different from median 54.3% after IUMR (*p *< 0.0001) and from 69.8% in our study population (*p *< 0.05) [21,25]. On the contrary, there was no significant difference for the need of shunting within the first year of life between IUMR and our population. However, the average age at shunt placement was significantly higher after IUMR (mean 21.2 days) than in our study population (mean 16.0 ± 10.7 days; *p *< 0.05). Likewise, median age at shunt placement was significantly higher after IUMR as compared to historical controls (85 vs. 5 days; *p *< 0.01) [22,24].

### Arnold-Chiari malformation and tethered cord

ACM II was radiologically diagnosed in 57.1% of our patients, one patient presented with ACM I. The incidence of ACM II was not different from historical controls (75.7%) [15]. Among our patients 18 (41.9%) presented with tethered cord and only two (11.1%) of them had to undergo surgery for TC so far, which is significantly less than after IUMR (29.6%; *p *< 0.01) and less than for historical controls (32.4%; *p *< 0.001), whereas the latter two did not differ significantly [5,26].

### Quality of life

In our population 56.4% and in historical controls 59.2% were assisted walkers that ambulate most of the time, both of which are significantly different from 92.6% of the patients after IUMR (*p *< 0.0001) [5,18]. Likewise, 40.8% of historical controls and 41.0% of our study population were reliant on a wheelchair, which is significantly more than after IUMR (7.4%; *p *< 0.0001). CIC was regularly performed in 69.7% of our population and similarly in 84.5% of historical controls [5]. Conversely, all patients after IUMR but only 38% of historical controls showed detrusor overactivity, suggesting that IUMR is associated with a higher incidence of complete denervation of the external urethral sphincter and detrusor overactivity [27]. A similar proportion of patients in all three groups attended regular education, suggesting adequate intellectual development (64.1%, 76.7% and 63.4% for Leipzig, IUMR and historical groups, respectively) [5,18]. There was no significant difference between shunted and non-shunted patients for the attendance of regular education in our population (80% vs. 58.6%). After IUMR, 67% of the patients had normal cognitive language and personal-social skills, 20% had mild and 13% significant delays at the age of two years, 23% were at risk for (significant) learning disabilities, with the majority (85.7%) shunted [28,29].

## Discussion

Myelomeningocele is a congenital anomaly that affects about 1,500 infants per year in the US, of which recently more than 90% survive the first year of life and about 75% will reach adulthood [5]. Even though MMC is a non-lethal birth defect, it is the associated life-long morbidity that motivates clinicians all over the world to examine the value of fetal therapy. The aim of IUMR is to improve postnatal morbidity and in particular neurological outcome by reduction of secondary injury to the spinal cord [30]. This should be achieved by coverage of the spinal defect to stop CSF leakage and to prevent secondary damage, which in turn might allow normal brain development. The initial fetoscopical approach failed to show convincing benefit due to a high rate of perinatal deaths, which could be ruled out when standard neurosurgical closure of the defect was performed prenatally via a hysterotomy [30]. Since then, in about 400 cases of IUMR a reversal of hindbrain herniation, a decrease in shunt-dependent hydrocephalus and an improved leg function as compared to historical controls have been suggested [4]. Those results will be confirmed or rejected in the randomized controlled MOMS trial that was initiated in 2003 in three major centers for fetal surgery in the US to evaluate potential benefits after prenatal versus postnatal MMC closure. While keenly awaiting the results of the trial, we performed a retrospective analysis on patients from our referral center for MMC and reviewed the literature to compare the outcomes of MMC patients treated at our institution with published data from IUMR and historical controls.

Comparable to historical controls, mean gestational age at birth in our patients was 37.8 weeks and no prenatal deaths were recorded. In contrast, after IUMR, mean gestational age at birth was 34.6 weeks with 11.8% of the infants born before 30 weeks of gestation, both significantly different from historical controls and our findings [21-23]. Perinatal mortality after IUMR was 5.9% [24]. As Bruner *et al *stated in 2005, virtually all fetuses that underwent IUMR deliver preterm and more than 10% even before 30 weeks, thus risking major morbidity [23]. That prompts the question if the postulated benefits of IUMR on hindbrain herniation, shunt-dependent hydrocephalus and leg function justify the associated risks for mother and child when IUMR in turn may cause major morbidity and neonatal death due to preterm delivery in a condition that is usually non-lethal [24]. Lethal complications due to chorioamnionitis, placental abruption and preterm premature rupture of the membranes have been reported in particular for *endoscopic *IUMR, diminishing a fetoscopic approach for MMC in the late 1990s [30]. To what extent surgical risks and preterm delivery due to *open *IUMR contributes to major morbidity and neonatal death cannot be estimated from the available data but might be answered by the MOMS trial.

The second main outcome of the MOMS trial, besides neonatal death, is the need for shunting. As compared to historical controls, the rate of shunting within the first year of life was significantly lower in our cohort and after IUMR, whereas the latter two did not differ significantly. This observation is backed up by the findings of others that reported shunt rates of 78% (n = 203) and 43.3% (n = 293), respectively, for postnatally-managed patients [31,32]. One reason for the discrepancy in shunt rates might be the inconsistency in clinical criteria for shunting, suggesting that the criteria characterized for the MOMS trial should become generally accepted (Table 1).

Another important finding of our study was the discrepancy in the rate of assisted walkers and wheelchair users between our population/historical controls and IUMR, for which 92.6% (assisted) walkers and only 7.4% wheelchair users have been reported [18]. At first glance this highly significant difference encourages the efforts of IUMR. However, the mean age of the study population was 21.7 years for historical controls, 13.3 years for our population and only 67.0 months after IUMR. Our results strengthen the finding that mobility decreases from early childhood to the early teen years [33]. Bowman *et al *showed that the percentage of patients ambulating the majority of time decreased from 76% at 0-5 years to 46% at 20-25 years, with a flattening beyond 10 years [5]. Assuming a similar progression for children that underwent IUMR, less than 50% might continue to be assisted walkers in their teens.

We are aware of the drawbacks of the conducted study, which might be affected by the inhomogeneity of study designs, study populations, outcome measurements and treatment modality between centers as well as by changes over time. Thus, our retrospectively-collected data might be compared to results assessed in varying populations, suggesting the comparison of 'apples and oranges'. However, we performed a comprehensive review of the literature to identify the best matching study groups for IUMR and historical controls in the outcomes of our interest. Even though studies varied in their study periods and sites, study populations consisted of patients with similar levels of lesions, a factor known to importantly influence the clinical course in MMC. Therefore, we assume that our cohort, as well as the study population of included studies reflects representative cross-sections of the 'true' MMC population.

## Conclusions

Considering the limitations of the presented study and the data available today, a clear benefit of IUMR has not yet been proven. The implementation of a close interdisciplinary management following postnatal MMC closure results in satisfactory long-term outcomes of patients suffering from this defect. With regards to the well-known challenges of fetal interventions for both, mother and child, in particular preterm delivery, we actually do not consider the implementation of IUMR at our institution as imperative and the results of the MOMS trial should be awaited before initiating a complex intervention like IUMR.

## List of abbreviations used

ACM: Arnold-Chiari malformation; CIC: clean intermittent catheterization; GA: gestational age; IUMR: intrauterine myelomeningocele repair; MMC: myelomeningocele; MOMS: Management of Myelomeningocele Study; TC: tethered cord

## Competing interests

The authors declare that they have no competing interests.

## Authors' contributions

CG and SM conceived of the study. MW, GG and CG carried out the outpatient clinic and data collection. SM carried out data retrieval, performed the statistical analysis and drafted the manuscript. CG and HT helped to draft the manuscript. All authors read and approved the final manuscript.
